# Investigating microbial dynamics and potential advantages of anaerobic co-digestion of cheese whey and poultry slaughterhouse wastewaters

**DOI:** 10.1038/s41598-022-14425-1

**Published:** 2022-06-22

**Authors:** M. Abdallah, S. Greige, H. Beyenal, M. Harb, M. Wazne

**Affiliations:** 1 Civil Engineering, Lebanese American University, 301 Bassil Building, Byblos, Lebanon; 2 The Gene and Linda Voiland School of Chemical Engineering and Bioengineering, Washington State University, Pullman, WA USA

**Keywords:** Environmental biotechnology, Environmental sciences

## Abstract

Resource recovery and prevention of environmental pollution are key goals for sustainable development. It is widely reported that agro-industrial activities are responsible for the discharge of billions of liters of wastewater to the environment. Anaerobic digestion of these energy rich agro-industrial wastewaters can simultaneously mitigate environmental pollution and recover embedded energy as methane gas. In this study, an assessment of mono- and co-digestion of cheese whey wastewater (CWW) and poultry slaughterhouse wastewater (PSW) was conducted in 2.25-L lab-scale anaerobic digesters. Treatment combinations evaluated included CWW (R1), PSW (R2), 75:25 CWW:PSW (R3), 25:75 CWW:PSW (R4), and 50:50 CWW:PSW (R5). The digestion efficiencies of the mixed wastewaters were compared to the weighted efficiencies of the corresponding combined mono-digested samples. R4, with a mixture of 25% CWW and 75% PSW, achieved the greatest treatment efficiency. This corresponded with an average biodegradability of 84%, which was greater than for R1 and R2 at 68.5 and 71.9%, respectively. Similarly, R4 produced the highest average cumulative methane value compared to R1 and R2 at 1.22× and 1.39× for similar COD loading, respectively. The modified Gompertz model provided the best fit for the obtained methane production data, with lag time decreasing over progressive treatment cycles. PCoA and heatmap analysis of relative microbial abundances indicated a divergence of microbial communities based on feed type over the treatment cycles. Microbial community analysis showed that genus *Petrimonas* attained the highest relative abundance (RA) at up to 38.9% in the first two cycles, then subsequently decreased to near 0% for all reactors. *Syntrophomonas* was highly abundant in PSW reactors, reaching up to 36% RA. *Acinetobacter* was present mostly in CWW reactors with a RA reaching 56.5%. The methanogenic community was dominated by *Methanothrix* (84.3–99.9% of archaea). The presence of phosphate and *Acinetobacter* in CWW feed appeared to reduce the treatment efficiency of associated reactors. Despite *Acinetobacter* being strictly aerobic, previous and current results indicate its survival under anaerobic conditions, with the storage of phosphate likely playing a key role in its ability to scavenge acetate during the digestion process.

## Introduction

Cheese whey wastewater (CWW) and poultry slaughterhouse wastewater (PSW) are major point sources of agro-industrial pollution if not properly treated. The global annual generation of cheese whey and poultry slaughterhouse wastewaters is estimated at 158 and 984 billion liters, respectively^1^. Most of this wastewater is discharged to the environment without treatment or is directed into community sewerage systems^2,3^. Both wastewaters are characterized by a high organic content, which may require significant energy input if treated using conventional methods such as the aerobic activated sludge process. Alternatively, the high organic load can be converted to energy as methane via anaerobic digestion. Based on this, the use of anaerobic digestion to treat both wastewaters is aligned with global initiatives focusing on renewable energy and circular economy^4–6^. The amount of recoverable energy in cheese whey and poultry slaughterhouse wastewaters is estimated at 24,500 and 12,200 GWh/year, respectively, or about 11% of the total energy contained in all agro-industrial effluents^1^.

Cheese whey is comprised mostly of biodegradable protein and carbohydrates with COD values up to 100 gCOD/L. However, anaerobic treatment of cheese whey could encounter challenges because of its low alkalinity and the high ratio of carbohydrates which could promote the growth of acid forming bacteria^2,7^. Moreover, cheese whey is reported to contain large amounts of salinity resulting from the manufacturing process, which could reach up to 8000 µS/cm^2,7,8^. This high salinity content could result in reduced digestion efficiency. Zhang et al. reported a hormetic pattern for the effect of salinity on the digestion of kitchen waste^9^. A salt concentration less than 4 g/L NaCl enhanced treatment by anaerobic digestion, whereas greater levels inhibited key bacterial enzymes which resulted in reduced methane yield efficiency. Similarly, Zhao et al. reported that low concentration of NaCl increased hydrolysis and acidogenesis but inhibited methanogenesis for anaerobic digestion of food waste, with both processes severely inhibited at high NaCl levels^10^.

Chicken slaughterhouse wastewater is rich in lipids and proteins with a high fraction of insoluble COD attributable to lipids^11^. The non-soluble COD fraction is reported to impede biological treatment. The hydrolysis of lipids during the anaerobic digestion process is reported to produce long chain fatty acids (LCFA), which can also have inhibitory effects during anaerobic digestion^11–14^. Salminen and Rintala reported that LCFA degradation is a limiting step in anaerobic degradation because of the slow growth of associated bacteria and the need for low H_2_ partial pressure^13^. LCFA are also reported to be toxic to acetogens and methanogens during anaerobic digestion. Their toxicity stems from their ability to adsorb to microbial cell surfaces. However, various substances are reported to mitigate LCFA inhibition, such as bentonite because of its flocculating capacity, or calcium from multiple sources because of its ability to form precipitates. In addition to LCFAs, various factors were reported to affect the performance of the anaerobic treatment of slaughterhouse wastewater such as free ammonia, volatile fatty acids (VFAs) and sulfate concentrations. High concentration of ammonia, resulting mainly from protein hydrolysis, and accumulation of VFAs caused a failure in the anaerobic digester treating these types of wastes and wastewaters due to inhibition of the microbial growth, thus decreasing the biogas production^13,15–19^. Excess amount of sulfate was also reported to reduce the performance of anaerobic digesters and impact the methane yield^18,20^.

Anaerobic digestion is a mature technology that has been used to convert organic waste to innocuous material and energy rich biogas. However, mono-digestion (digestion of a single feedstock) could suffer from challenges attributed to imbalances resulting from the use of a single substrate. Anaerobic co-digestion (digestion of multiple wastes) can mitigate problems encountered during mono-digestion^2,21–23^. The improvement could be due to the adjustment of C/N ratio, dilution of the concentration of toxic elements, or supplementation of missing micronutrients. Co-digestion of cheese whey and poultry slaughterhouse wastewaters could have specific advantages towards improving the overall digestion process and methane yield. Cheese whey wastewater are reported to have high calcium concentrations which could serve to mitigate the inhibition caused by LCFA in poultry slaughterhouse wastewater^24,25^. Moreover, the high concentration of NaCl in the cheese whey can help to solubilize particulate organic matter in the poultry slaughterhouse wastewater^10^. Lastly, the co-digestion process could dilute the concentration of NaCl in the cheese whey and potentially improve the treatment efficiency and methane yield.

Even though CWW and PSW appear to have complementary characteristics relating to anaerobic co-digestion, no studies have investigated such treatment while directly focusing on microbial analysis to elucidate the basis of improvement. This is especially important since performance efficiency is dependent on the complex syntrophic interactions among microbial communities whose diversity, synergism and competition are highly affected by operational parameters and compositions^26^. This study investigated the efficiency of mono-digestion and co-digestion of CWW and PSW in batch tests over four treatment cycles. High-throughput 16S rRNA sequencing of the microbial communities of reactors was conducted to delineate specific microbial development patterns that could aid in the understanding and enhancement of the treatment process.

## Materials and methods

### Substrate collection and preparation

Two high strength agro-industrial wastewaters, poultry slaughterhouse wastewater (PSW) and cheese whey wastewater (CWW), were obtained from Bekaa, Lebanon. Seed sludge was obtained from an anaerobic digester at the Bkassine wastewater treatment plant located in Saida, Lebanon. The collected wastewaters and seed sludge were manually sieved with a mesh of 1.6 mm size before homogenization. The wastewaters were manually grinded using a household blender (KitchenAid, Michigan, USA), homogenized, and stored in plastic 1-L bottles at − 20 °C until used. The sludge biomass had an approximate VSS value of 17.1 g VSS/L.

### Experimental setup

Five 5-L continuously stirred tank reactors (CSTR) (CHEMGLASS, New Jersey, USA) were inoculated with the seed sludge and the substrate mixtures to achieve working reactor volumes of 2.25-L and were placed in a water bath operating at mesophilic conditions (37 °C) for 63 days. The experiment was executed with an initial acclimation period of 22 days (C0), followed by three cycles of 10 days each (C1 to C3) and a fourth cycle for 6 days (C4). The phased cycles were used to monitor and compare the development of microbial dynamics and the resulting methane yield for different feed ratios under similar operational conditions. Two reactors were operated in mono-digestion mode. For the remaining three reactors, substrate mixtures (VS ratio basis) (CWW:PSW) were 75:25, 25:75 and 50:50. Each reactor was seeded to maintain a 1:1 VS substrate to inoculum ratio and an OLR of 1 g COD/L/day. At the end of each cycle, 0.25-L reactor content was removed and replaced with an equivalent volume of substrate. Biogas production, pH, and VFAs were monitored on a daily basis, while COD and VSS were analyzed at the start and end of each feeding cycle. Each reactor was purged with N_2_ for 15 min after each feeding.

### Performance criteria and analytical methods

The performance of the different reactors was assessed based on biodegradability and synergy. Biodegradability is defined as the ratio of the specific methane production over the theoretical methane production of the substrates. Synergy is defined as the ratio of the co-digestion efficiency of a mixed substrate over the corresponding weighted digestion efficiency of equivalent substrate using mono-digestion. The digestion efficiency is defined as the ratio of the measured methane produced over the methane potential based on the COD of added substrate (Supplementary Information).

The following parameters were determined in this study: Total Solids (TS), Volatile Solids (VS), Total Suspended Solids (TSS), Volatile Suspended Solids (VSS), Total Chemical Oxygen Demand (tCOD), Soluble Chemical Oxygen Demand (sCOD), protein, carbohydrates, lipids, reactive phosphate, inorganic phosphate (meta-, poly-), total phosphate, ammonia (NH_3_), Total Organic Carbon (tTOC), Soluble Total Organic Carbon (sTOC), Total Nitrogen (tTN), Soluble Total Nitrogen (sTN), Total Kjeldahl Nitrogen (tTKN), Soluble Kjeldahl Nitrogen (sTKN), acetic acid, propionic acid, butyric acid, methane gas (CH_4_), carbon Dioxide gas (CO_2_), K^+^, Na^+^, Mg^2+^, Ca^2+^, Cl^−^, NO_2_^−^, NO_3_^−^, SO_4_^2−^. Cl^−^, NO_2_^−^, NO_3_^−^, SO_4_^2−^. The detailed description of the corresponding analytical techniques is included in the Supplementary Information.

### Kinetic modelling

Two mathematical models were used to fit the methane production data: the modified Gompertz equation and the first order kinetic model. Nonlinear regression was used to determine the kinetic parameters associated with the two models using IBM’s SPSS Statistics 26 software.

#### Modified Gompertz model

This empirical model is based on the concept that methanogens' growth follows a non-linear trend line consisting of a lag phase followed by an exponential phase^27^. It is expressed in Eq. (1) as:

1 $${\text{Q }} = {\text{Q}}_{0} \times {\text{e}}^{- {\text{e}}^{\frac{{\text{P}}_{\text{m}} \times {\text{e}}}{{\text{Q}}_{0} } \times \left( \uplambda - {\text{t}} \right) + 1}} ,$$

where Q_0_ is the maximum possible methane potential (mL-CH_4_/g COD), P_m_ is the maximum methane production rate (mL-CH_4_/g COD/d), λ is the lag phase period (d) and Q is cumulative methane potential at a given time t (mL-CH_4_).

#### First order kinetic model

The first order kinetic model assumes that the rate-limiting step in methane production is the hydrolysis rate of the substrate denoted by a rate constant. This model only considers the exponential phase of the methane production^28^. Equation (2) shows the model as follows:

2 $${\text{Q }} = {\text{Q}}_{0}\times (1 - {\text{e}}^{-{\text{kt}}}),$$

where Q_0_ is the maximum possible methane potential (mL-CH_4_/g COD), Q is cumulative methane potential at a given time t (mL-CH_4_), and k is the first order hydrolysis rate (day^−1^).

### Microbial community analysis

For downstream PCR and sequencing, genomic DNA was extracted using the DNeasy PowerSoil Kit (Qiagen, USA) according to the manufacturer’s protocol. DNA was eluted in Tris–EDTA (TE) buffer. DNA concentration and purity were checked using a Nanodrop spectrophotometer. The extracted DNA was stored at − 20 °C until used. DNA samples were analyzed by Novogene, Singapore. PCR amplification of the V3–V4 region of the 16S rRNA gene using the primer set 341F (5ʹ-CCTAYGGGRBGCASCAG-3ʹ) and 806R (5ʹ-GGACTACNNGGGTATCTAAT-3ʹ) with barcodes (470 bp) was followed by library preparation and sequencing on a paired-end Illumina NovaSeq 6000 sequencing platform to generate 250 bp paired-end raw reads. For sequencing data processing and analysis, the Mothur tool suite on the Galaxy server was used, with Silva 132 reference database for alignment and Ribosomal Database Project (RDP) trainset18_062020 reference taxonomy for classification^29^. A heat map was generated from the relative abundance of OTUs using excel. Beta diversity was assessed by clustering of samples into groups within a principal coordinate analysis (PCoA). For the comparison of average relative abundances of sample sets, an independent (unpaired) two-tailed t-test was used. The representative sequences obtained were compared with all 16S rRNA sequences available from the National Centre for Biotechnology Information (NCBI) using the nucleotide Basic Local Alignment Search Tool (BLAST) program.

## Results and discussion

### Substrate characterization

The characterization data of the CWW and PSW samples are listed in Table S1 (Supplementary Information). Both samples exhibited nearly similar total COD and VS values; however, they differed in terms of other constituents. The total solids (TS), volatile solids (VS), total COD (tCOD), and soluble COD (sCOD) for the cheese whey sample were 9.8%, 85.9% of TS, 91,300 mg/L, and 81,500 mg/L, respectively. The corresponding values for the PSW were 7.9%, 95.8% of TS, 86,500 mg/L, and 16,800 mg/L, respectively. The CWW sample was comprised mostly of proteins and carbohydrates (52.3%TS and 33.1%TS, respectively) while having negligible lipid content (0.37%TS). Conversely, the PSW sample contained mostly lipid fractions (63.6%TS) while having low carbohydrates (6.24%TS). Studies conducted by Lu et al. have shown that higher carbohydrates content could contribute to lower methane production while higher lipids content tend to increase methane production^30^. In addition, Niefla et al. suggests that the high lipid content can contribute to higher SMP values compared to protein and carbohydrates yet may exhibit lower kinetics in terms of its hydrolysis^31^. However, high lipid content may induce inhibition to acidogenesis and methanogenesis due to higher long chain fatty acids production which can adsorb to microbial cells and inhibit their metabolic activity.

The cheese whey sample exhibited greater concentrations of NaCl salt as evidenced by the high concentration of Na^+^ and Cl^−^ at 3011 and 5195 mg/L, respectively. The corresponding Na^+^ and Cl^−^ concentration for the PSW sample were 409 and 68 mg/L, respectively. Similarly, the cheese whey sample exhibited greater concentrations of Ca^2+^, K^+^, Mg^2+^, NO^2−^, and SO_4_^2−^ at 556, 1482, 378, 30, and 149 mg/L, respectively, whereas the corresponding concentrations of the PSW sample were 2, 151, 108, 3, and 32, respectively. The high salinity content could result in reduced digestion efficiency^9^. On the other hand, the high salt concentration could help in the solubilization of the carbohydrates and proteins^10^. The cheese whey sample also had greater concentrations of ortho-phosphate, organic phosphate, and polyphosphate at 1210, 1245, and 425 mg/L, whereas the corresponding values for the PSW are 106, 22 and 0 mg/L, respectively.

The cheese whey, PSW, and the seed sludge have pH values of 5.7, 6.57, and 8.35, respectively. The relatively low pH value of the cheese whey is reported due to the low alkalinity of such wastewaters, which could result in reduced digestion efficiency^32^. The C/N ratios of the five reactors ranged between 33/1 and 21/1 for reactors R1 (100%CWW) and R2 (100%PSW), respectively. The C/N ratios for all reactors appear to fall very close within the recommended range of 20/1 to 30/1^33^.

### Methane production

Theoretical methane potential is used in the assessment of the expected cumulative methane production and determination of the biodegradability of the substrate. The values obtained as per Eq. (S1) for CWW and PSW were 401 mL-CH_4_/gVS_added_ and 799 mL-CH_4_/gVS_added_ which correspond to 396.92 mL-CH_4_/gCOD_added_ and 375.7 mL-CH_4_/gCOD_added_. Similar values were reported for similar wastewaters^32,34–36^. Based on the COD values of the added substrates, the absolute theoretical methane potential (TMP) for the different mixtures and singular substrates were close with values of 9018 mL, 8544 mL, 8218 mL, 8554 mL, and 8169 mL for R1 to R5, respectively.

**Figure 1:**
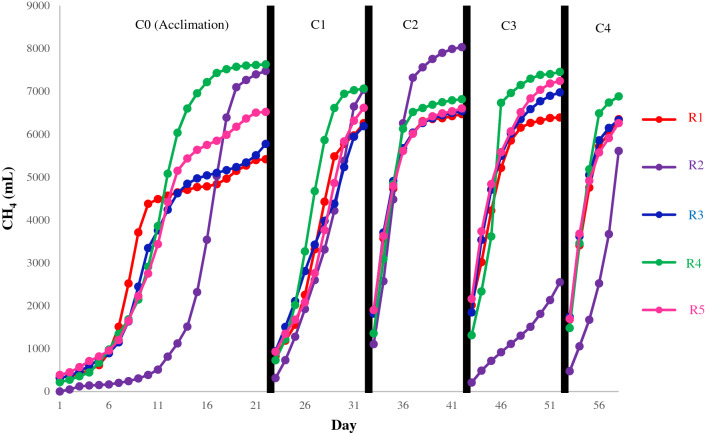
Cumulative methane production during the acclimation and 4 cycles for each of the reactors: R1 (100% CWW), R2 (100% PSW), R3 (75% CWW & 25% PSW), R4 (25% CWW & 75% PSW) and R5 (50% CWW & 50% PSW).

The biogas production was monitored for all reactors on a daily basis. The cumulative and daily methane production are shown in Fig. 1 and Fig. S1 (Supplementary Information). The acclimation cycle ended after 22 days, at which the cumulative methane yield for R1, R2, R3, R4 and R5 was 259, 397, 283, 395 and 328 mL/gCOD_added_. R2 and R4 showed the highest methane yields. R1 exhibited the lowest yield among the different reactors, as per Fig. 1. However, in terms of daily methane production, R1 (100% CWW) exhibited the fastest methane production whereby it peaked the earliest (at 9 days), while R2 (100% PSW) registered the longest time to reach its peak (17 days). The long lag time observed for R2 was reported for similar feed^12^. The co-digestion reactors exhibited peak methane productions that fell between the two mono-digestion reactors being closer to each end based on the percent composition.

By the end of the 22-day period, all reactors exceeded 80% of their methane potentials and reached a plateau as seen in Fig. 1. The fast production of biogas by the reactor fed with CWW could be attributed to the readily biodegradable matter of the waste, as it contained mostly soluble material, while that of the PSW mainly constituted complex particulate matter which led to its longer lag time^37^.

After the acclimation phase, each reactor was fed with the same waste combination at the same loading for a fixed period of time (10 days for the first 3 phases and 6 days for the last phase) to observe any improvement in terms of biogas production. Daily methane productions demonstrated in Fig. S1 showed variations in terms of the per cycle development of the methane production as well as differences between the reactors themselves. Lag time for daily peaks were observed in the acclimation cycle, increasing with the lipid content of the wastewater^35^. As the cycles progressed, the maximum daily peaks were observed much earlier, except for R2 which developed a lagged production in the 3rd cycle. R2 performance deteriorated during the third cycle whereby no peaks were observed, and a rising trend started during the fourth feeding cycle indicating a significant lag time. This result could suggest that digestion of 100% PSW in R2 experienced inhibition during the latter feeding cycles. Figure S2b shows notable pH variation in cycle 3 for R2, where the pH drops throughout the digestion period to below 7 as opposed to the trend of the other reactors where the pH increases above 7 and stabilizes. This could be correlated to the accumulation of VFAs in the system, as seen in Fig. S3b where there was a rise in VFA levels around the 6th day of operation compared to the other reactors where the VFAs level was nearly depleted. Furthermore, Cirne et al. investigated the effects of lipid rich wastewaters on the anaerobic digestion process. It was found that despite the high methane content for lipids, digestion inhibition can occur for high content (i.e., greater than 47% TS lipids)^12^. This was attributed to the buildup of long-chain fatty acids (LCFAs) as well as the liquefaction of the complex lipids onto cell membranes during hydrolysis^38^.

As for the total methane produced in each cycle for the reactors, Table 1 shows that R4 had produced the highest total methane at the end of the cycle compared to all the other reactors, being on average for all cycles 21.62% and 38.54% higher than CWW and PSW mono-digestion experiments. Furthermore, R4 specific methane yield was significantly greater than the specific methane yield for R3 and R5 for all cycles (*P* < 0.05). For the 2nd feeding cycle, where R2 had a higher production, this could be attributed to residual substrates from the previous cycle as the biodegradability of R2 in the first cycle was less than that for the acclimation cycle by 6%. However, the difference became significant in the 3rd and 4th cycles due to the inhibition occurring in R2. On the other hand, R1 consistently had the lowest amount of produced methane compared to the co-digestion reactors and R2, yet it produced methane with the lowest lag times.

**Table 1:** Summary of the biodegradability results.

	Phase	R1	R2	R3	R4	R5
Biodegradability (%)	Acclimation	60.1	87.5	70.3	89.2	79.8
	Cycle 1	69.4	82.4	75.3	82.6	81.0
	Cycle 2	71.8	94.0	79.5	79.7	80.8
	Cycle 3	70.9	29.9	84.9	87.1	88.7
	Cycle 4	70.4	65.7	77.1	80.5	76.6

CWW in our study mainly consisted of carbohydrates and proteins which are relatively easier to biodegrade than lipids. The presence of easily biodegradable carbohydrates (such as lactose and glucose) and proteins, in addition to their high solubility, may have resulted in facilitated hydrolysis which was reflected in the lower lag time^37,39^. Carvalho et al. and Kassongo et al. reported that lactose is the main fermentable carbohydrate in CWW; however, not many microbial communities are adapted to its degradation^32,34^. On the other hand, Zhao et al. highlighted the importance of salinity concentration in which high levels of salinity (greater than 10 g/L NaCl) could inhibit acidogenesis and methanogenesis; thus, reducing the possible amount of methane produced^10^. CWW exhibited high salinity content as reflected as per Table S1.

### Biodegradability

The biodegradability indices for all reactors and cycles are shown in Table 1, and the equation is presented in the Supplementary Information per Eq. (S2). Despite the similar absolute methane potential of the two substrates (due to close COD values)—and subsequently the co-digestion mixtures—the experimental methane yield significantly varied between the five different batches which may indicate the presence of a non-biodegradable fraction in the wastes. R1 had the lowest biodegradability with a maximum value of 72%. R2 exhibited better biodegradability reaching 94% during the 2nd feeding cycle. However, this value drops for R2 by the 3rd feeding cycle to 30% indicating digestion inhibition as evidenced in the VFA buildup shown in Fig. S3b. R3 and R5 biodegradability improved beyond the acclimation cycle whereas R4 biodegradability decreased in the first two feeding cycles then increased to 87% by cycle 3. The results shown in Table 1 prove that R4 had the highest biodegradability values throughout all phases (considering cycle 3’s error for R2) with values of 89.21%, 82.61%, 79.70%, 87.14% and 80.46% for each cycle respectively. This indicates that the co-digestion mixture of 75:25 PSW:CWW used in R4 was able to amend the complexity of the hydrolysis phases of the anaerobic digestion to obtain higher methane values within a shorter frame of time while mitigating any inhibitory effects that can hinder digestion compared to the mono-digestion processes. Results in Fig. S3 show that the co-digestion systems were able to overcome acidogenesis with no significant buildup of VFA or drop in pH (Figs. S2, S3).

Some of the reported causes for the reduced digestion efficiency of the cheese whey feed include low alkalinity, unfavorable C/N ratio, or salinity. The pH values of CWW reactors at about 7 or greater indicates that pH was not the determining factor in the reduced efficiency. Similarly, the C/N ratios of all CWW reactors are close within the recommended C/N ratios of 20/1 to 30/1, which precludes this factor for any reduced efficacy. Moreover, the NaCl concentration in the CWW appears not to be a major factor in the reduced efficiency. Even though the Na^+^ concentration in the CWW feed is 3011 mg/L (equivalent to 7.66 g/L NaCl) appear to exceed the threshold of 4 g/L needed to result in inhibition; however, the CWW was diluted by the seed sludge to much less levels. This indicates that other factors could be responsible for the reduced efficiency including unfavorable microbial development.

Conversely, the PSW biodegradability factors were significantly greater with increased PSW ratios in the feed except for the apparent inhibition for R2 in cycle 3 due to VFA build up. The lag time exhibited in the acclimation cycle appears to decrease with the progression of the treatment cycles. It appears that R4 with feed distribution of 75:25 PSW:CWW was optimal in this study. Even though salt or calcium in the cheese whey samples may have played key roles in enhancing the treatment efficiency, no conclusive evidence was traced to either. Future controlled studies could focus on the direct impact of salt and calcium addition on the treatment efficiency of PSW.

### Treatment efficiency and synergistic effects

**Figure 2:**
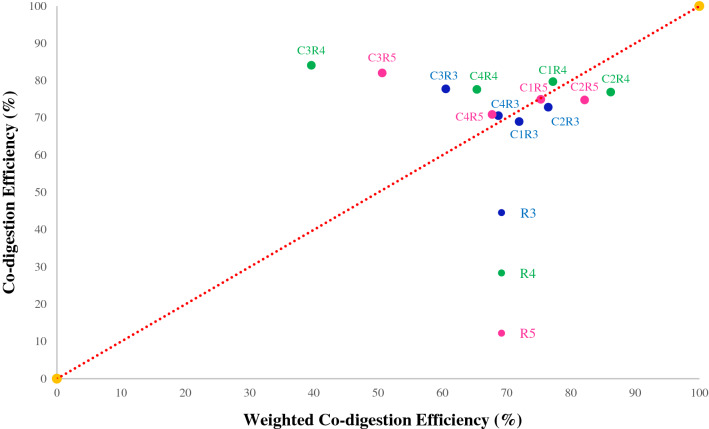
Co-digestion efficiency of mixed wastewater versus the weighted efficiencies of the corresponding combined mono-digested samples with respect to the identity line (Y = X, dotted red line) for the co-digestion reactors during the 4 cycles after acclimation: R3 (75% CWW & 25% PSW), R4 (25% CWW & 75% PSW) and R5 (50% CWW & 50% PSW).

To assess the synergistic effects of co-digestion, the treatment efficiency for mixed substrates was compared to the corresponding independent weighted efficiency by each substrate. Figure 2 demonstrates the relationship between the co-digestion efficiency and the weighted co-digestion efficiency to determine whether synergistic effects were present within the experiment with respect to the identity line (Y = X). A value which plots above the identity line indicates synergism, whereas a value which plots below the line indicates reduced efficiency. As shown in Fig. 2, no significant synergism was observed in the first two cycles (C1 and C2). On the other hand, significant synergisms were observed in cycle three (C3) for R3 and R5. However, R4 registered the greatest synergism as compared to the other reactors, being furthest above the identity line in cycles 3 and 4. The synergism observed in the third cycle (C3) may be was impacted by the limited inhibition in R2 in that cycle.

### Kinetic modelling

Kinetic models are important tools to quantify, predict and understand synergistic improvements in biogas production. Key factors obtained from these specific rate-limiting models include digestion rates, lag times and production rates^40^. In this study, two of the most applicable models in anaerobic digestion were used to simulate the cumulative biogas production data, the modified Gompertz model and first order kinetic model (Table S2). A summary of reported kinetic parameters for substrates of relatively similar composition to the ones in this study is provided in Table S3. The modified Gompertz model provided the best fit for our data with r^2^ values higher than 0.98 for all reactors and cycles except for R4 (3rd cycle) and R2 (3rd and 4th cycle) (r^2^ = 0.96, and r^2^ = 0.97 and non-applicable fit, respectively). During the acclimation phase, all reactors showed a lag time (λ) with R1, R3 and R5 having the least value of about 5 days. However, in the cycles that followed, the lag time decreased to nearly zero days beyond the 1st feeding cycle for all reactors except for R2 where these values increased to 2 days during the 3rd cycle, indicating possible complications in the digestion process which can be attributed to LCFA presence^12^. Other studies also reported that the modified Gompertz model provided a good fit for the experimental cumulative methane data^31,37^. Lag times in the anaerobic digestion of cheese whey products reached up to 2 days as reported by Bella and Rao, which can be attributed to the lack of biomass adaptation to the substrate and complications in the digestion of complex whey proteins^41^. Studies performed on slaughterhouse wastes with high lipid content have shown that lag times of up to 3 days might occur due to possible LCFA and VFA build-up^42,43^. However, R2 and R4 had much higher lag times (13 days and 6 days respectively); yet the lag time in R4 decreased to zero beyond the 1st cycle. These results demonstrate that CWW addition at different proportions could facilitate the digestion process due to its relatively readily biodegradable structure^37^.

The maximum production rate P_m_ demonstrated a relatively increasing trend with cycle progression except for R2 showing fluctuations in addition to model inapplicability in the 3rd cycle. R4 had the highest production rates (with similar values compared to R2 in cycle 2) as compared to the other reactors which could indicate a facilitated metabolism of the PSW wastewater with the added proportion of CWW^31,37^. Maximum methane production rate values vary for the same substrate composition (Table S3). The reported values for yogurt whey, cheese whey and a substrate mixture of organic fraction municipal solid waste with biological sludge (having organic composition similar to CWW) were 13.9, 7.52 and 35.1 mL-CH_4_/gVS/day, respectively^31,37,41^. As for slaughterhouse wastes, these values ranged from 22.9 to 87.4 mL-CH_4_/gCOD/day^42,43^. Production rates for R1 and R2 reached 77.4 mL-CH_4_/gCOD/day and 55.5 mL-CH_4_/gCOD/d by the 4th cycle (77.4 mL-CH_4_/gVS/day and 116.5 mL-CH_4_/gVS/d respectively based on COD/VS ratios in Table S1) which are greater than those reported in the literature.

The first order model was applied to fit the biogas data and evaluate the rate-limiting constant k. However, this model yielded a poor fit especially during the acclimation and 1st cycle with r^2^ values ranging between 0.729 and 0.969 for all reactors. The first order model accounts for the exponential phase of the biogas production without incorporating the lag phase which could explain the low r^2^ values obtained^37,40^. By removing the data points corresponding to the lag phase, an improved fit was observed with r^2^ ranging between 0.94 and 1.00 for all reactors and cycles. The rate constant (k) values show an increasing trend with cycle progression (beyond cycle 1) except for R2 due to a non-applicable fit in the 3rd and 4th cycles because of the hindered biogas production. The k values reached stable levels beyond the 2nd cycle for all reactors except R2. Despite R4’s better performance, the rate constant was the highest only in the 2nd cycle (0.64 day^−1^) while the other reactors containing higher proportions of CWW showed higher k values in the following cycles. Literature reported typical values for hydrolysis rates depending on the substrate composition with higher values for protein rich substrates compared to lipid rich substrates^35^. Neves et al. reported hydrolysis rate constants using first order kinetics of 0.12 day^−1^ and 0.24 day^−1^ for lipid and protein rich wastes, respectively, whereas Nielfa et al. reported a value of 0.23 day^−1^ for yogurt whey^31,35^. The composition of the aforementioned wastes was relatively similar to that of R2 (lipid-rich) and R1 (protein rich). However, R1 and R2 have shown greater (k) values than those reported, except for the value obtained for R1 during the acclimation cycle. The obtained results demonstrate that the presence of CWW in the reactors, especially with those containing PSW, could facilitate the anaerobic degradation of challenging substrates through altering microbial community dynamics in a way that would enhance microbial metabolism, syntrophy and kinetics^37^.

### Microbial community

**Figure 3:**
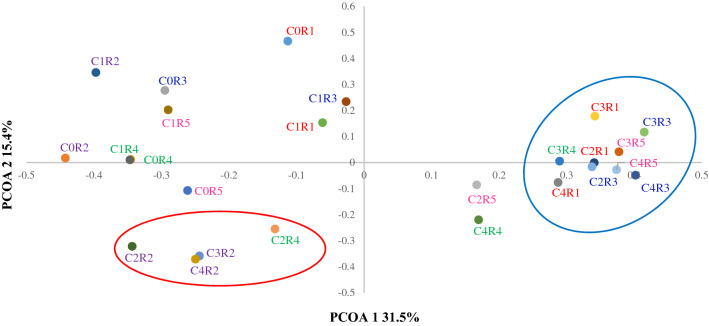
Principal coordinate analysis (PCoA) of microbial activity similarity for the five reactors samples based on thetaYC similarity distance matrix of genus-based sequence clusters. The first letter indicates cycle number (C), whereas the second letter indicates reactor number (R).

**Figure 4:**
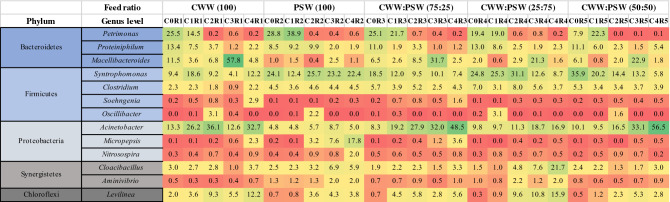
Heat map representing the relative abundance of the most abundant bacterial genera in the five reactors (R1, R2, R3, R4, and R5) through the process of anaerobic digestion (C0, C1, C2, C3, and C4) (≥ 2% in at least one sample).

**Figure 5:**
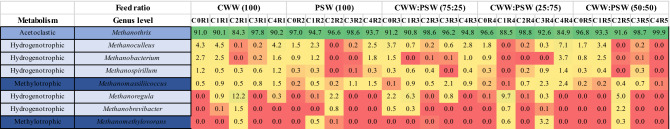
Metabolic association of archaeal genera observed as well as the relative abundance within reactors’ samples (R1, R2, R3, R4, and R5) at the end of each operational cycle (C0, C1, C2, C3, and C4) of the experiment (≥ 1% in at least one sample).

The PCoA of microbial activity similarity for the five reactors samples is shown in Fig. 3. Heatmaps (Figs. 4, 5) of bacterial and archaeal relative abundances (RA) with a cut-off of 2% and 1% or greater of the reads in at least one sample, respectively, was used in the analyses of the microbial community changes.

The reactor treating only PSW (R2) showed significantly lower relative abundances of the genera *Petrimonas* and *Proteiniphilum* as feeding cycles advanced (unpaired t-test, *P* ≤ 0.05). On the other hand, *Cloacibacillus, Levilinea, Aminivibrio, Nitrosospira,* and *Micropepsis* were identified as having significantly higher RA in R2 (unpaired t-test, *P* ≤ 0.05). A significant increase in the abundance of *Methanomassiliicoccus* was also seen in R2 as cycles progressed (unpaired t-test, *P* ≤ 0.05).

The major shift in the microbial community was observed when comparing the red and blue clusters. These clusters showed a clear separation of R2 and samples belonging to R1, R3, and R5. The latter showed a remarkably higher RA of *Macellibacteroides, Acinetobacter*, and *Soehngenia* in R1, R3, and R5 fed by CWW alone or in combination with different ratios of PSW (unpaired t-test, *P* ≤ 0.05). Conversely, *Syntrophomonas*, *Aminivibrio*, and *Micropepsis* were more abundant in the lower left part of the ordination (reactor R2) (unpaired t-test, *P* ≤ 0.05). The archaeal community showed a significant increase in the RA of *Methanospirillum* (unpaired t-test, *P* ≤ 0.05) in R1, R3 and R5 (*P* values: Table S4 Supplementary Information).

#### Archaeal structure

The heatmap of relative abundance of archaea (greater 1%) shows that *Methanothrix*, had extremely high abundance (84.3–99.9%), followed by a variety of hydrogenotrophic methanogenic genera such as *Methanoculleus*, *Methanoregula*, *Methanobacterium*, *Methanospirillum*, and *Methanobrevibacter* (Fig. 5). The majority are known to utilize CO_2_, H_2_, and formate to produce CH_4_^44–46^. *Methanospirillum* had a very weak growth with formate, and *Methanobrevibacter* requires an acidic environment and acetate for growth on H_2_ and CO_2_. Two methylotrophic methanogens, *Methanomassiliicoccus* and *Methanomethylovorans*, were also found in our samples. These strains reduce methanol and other methylated compounds to produce methane^47,48^. This domination of methane production through the acetoclastic route over the hydrogenotrophic and methylotrophic ones was reported previously in anaerobic bioreactors^27,49^. *Methanothrix* showed a significantly higher RA in R2 when compared to R1. Conversely, *Methanospirillum* showed a significant lower RA (unpaired t-test, *P* = 0.05 and *P* = 0.01). The latter, as well as *Methanomassiliicoccus*, had higher RA in R1 when compared to R5 (unpaired t-test, *P* = 0.02 and *P* = 0.04).

#### Bacterial community composition and dynamics

The bacterial community structure and composition are presented in the generated heatmap (Fig. 4). A consortium of Bacteroidetes, Firmicutes, Proteobacteria, Synergistetes, and Chloroflexi were dominant in all bioreactor samples. The overall dominance of these phyla was described previously^50–53^. To further elucidate the functional adaption of the microbial community, thirteen bacterial genera were characterized as highly abundant in our reactors with a threshold of 2% of the reads in at least one sample and with a percentage of identity above 94%. Six bacterial genera, *Acinetobacter*, *Syntrophomonas*, *Petrimonas*, *Macellibacteroides*, *Proteiniphilum*, and *Levilinea*, were highly present in all studied samples.

*Petrimonas* and *Proteiniphilum* belonging to the *Dysgonomonadaceae* family of the *Bacteroidetes* phylum, were highly abundant at the beginning of the process. The relative abundance of *Petrimonas* was highest (38.9%) in the startup cycles (C0 and C1), then it decreased drastically to reach 0% in the rest of cycles in all reactors. *Proteiniphilum* had the highest relative abundance in the initial cycle C0 of all reactors, then decreased. *Petrimonas* is capable of fermenting carbohydrates to produce acetate, CO_2_, and H_2_. *Proteiniphilum* produces acetate and CO_2_ as end products of pyruvate’s fermentation from proteinaceous materials^54,55^.

*Macellibacteroides* is another fermentative-acetogenic bacteria which metabolizes monosaccharides and disaccharides with main fermentation products of lactate, acetate, butyrate and isobutyrate^56,57^. This genus peaked during cycle three (C3) of all reactors followed by a drastic decrease in cycle four (C4). R2 showed a RA ranging from 0.4 to 2.5% with an alternative increase and decrease throughout the experiment. Interestingly, the abundance increases in the rest of the reactors fed by CWW alone (57.8%) or in combination with different ratios of PSW (31.7% with ratio 75:25, 21.3% with ratio 25:75 and 22.9% ratio 50:50).

*Syntrophomonas* are capable of degrading long-chain fatty acids (LCFAs) through *β*-oxidation. Thus, these LCFA-degrading bacteria were highly enriched in the reactors treating wastewaters with high LCFAs content in order to convert them to acetate which was further catalyzed to methane^58^. *Syntrophomonas* was noticed to be highly abundant during anaerobic degradation of lipid rich waste i.e. PSW fed to reactors R2, R4 and R5 reaching about 36%. When it comes to reactors R1 and R3 (with CWW as predominant feed), the system maintained an abundance of this genus ranging between 4.1 and 18.5% RA. This was statistically significant when comparing its RA between R2 versus R1 and R3 (unpaired t-test, *P* = 0.01 each).

Chloroflexi was dominated by *Levilinea*, which increased moderately in abundance throughout the experiment with highest values during mono digestion of CWW and as well as co-digestion of this feedstock with PSW at a ratio of 25:75 (in R4) with no apparent trend. Carbohydrate and protein sources in both feeds likely contributed to its dominance, as it is known as a fermenter capable of converting sugars and amino acids into small molecules like H_2_, acetic, and lactic acids. Moreover, *Levilinea* is reported to be only slightly inhibited by salinity at concentrations up to 30 g NaCl/L^59^.

The phylum of Proteobacteria was represented by *Acinetobacter*, which is a non-fermentative bacteria known to use acetate as a carbon source and is unable to produce acids from carbohydrates^60^. Although *Acinetobacter* species are known to be strictly aerobic, their survival through the anaerobic digesters was widely reported^61–65^. In our findings, the RA of *Acinetobacter* increased across the course of the experiment with the highest values in reactors R1, R3 and R5, reaching up to 56.5% (digesters with 100%, 75% and 50% of CWW), and lower relative abundances in reactors R2 and R4 (≤ 18.7%) (100% and 75% of PSW). Statistically significant higher RAs were observed in reactors R1, R3 and R4 compared to R2 (unpaired t-test, *P* = 0.006 for R2 vs. R1 and 4, and *P* = 0.01 for R2 vs. R3).

The analysis of the *Acinetobacter* in the feed indicated a relative abundance of 7.8% and 0.5% for the cheese whey and poultry slaughterhouse wastewaters, respectively (Fig. S4). *Acinetobacter* has been shown to survive under anaerobic conditions when having stored phosphates^64,66^. The increase in the relative abundance of *Acinetobacter* in the CWW reactors over cycles is most likely due to accumulation from the feed. Hydrolysis of intracellular stored polyphosphate is reported to sustain *Acinetobacter* under anaerobic conditions. The measured concentrations of ortho-phosphate, organic phosphate, and polyphosphate in the CWW were 1210, 1245, and 425 mg/L, respectively, while the corresponding concentrations in the PSW were 106, 22, and ND mg/L, respectively. The high concentration of phosphate in the cheese whey most likely originated from the manufacturing process of cheese^25^. Under anaerobic conditions, *Acinetobacter* can utilize stored polyphosphate as energy source to take up short chain acids such as acetate and store them as polyhydroxybutyrate (PHB), whereas under aerobic conditions, it utilizes the stored PHB for growth and storage of phosphorus as polyphosphate^67^. Oehmen et al. reported that under anaerobic conditions, polyphosphate accumulating organisms (PAO) convert VFA to PHA using energy obtained from the hydrolysis of two stored polymers, polyphosphate and glycogen^68^. Polyphosphate is hydrolyzed to ortho-phosphate whereas glycogen is converted to PHA and CO_2_. Mino et al. reported that both polyphosphate and glycogen are needed for the uptake of organic substrate under anaerobic conditions. Polyphosphate supplies the energy, whereas glycogen supplies the needed reducing power^69^. It is worth noting that a significant amount of biogas consisting of almost 100% CO_2_ was released from the CWW reactors within the first 24 h after the start of the reactors, in proportion to the CWW wastewater fraction (Fig. S5). This supports the active presence of PAO in the CWW reactors in our study. The ratio of phosphorus released to acetate uptake (mol P/mol C) can reach up to 1.52^69^. This stoichiometric ratio is less than the ratio needed to explain the reduced CWW methane yield based on the COD and phosphate concentrations in the CWW feed. However, the early vigorous activity of the PAOs in the CWW reactors (such as *Acinetobacter*) may have negatively impacted the syntrophic development of the microbial dynamics and thus resulted in reduced efficiency.

## Conclusion

The co-digestion of the CWW and PSW feeds resulted in improved methane yield, likely due to their complementary characteristics which aided in balancing the anaerobic digestion process. R4 with feed distribution of 75:25 PSW:CWW was optimal in this study. It produced the highest average cumulative methane at 1.22× and 1.39× the values obtained for R1 and R2 for similar COD loading, respectively. It also achieved the greatest average biodegradability at approximately 84%. The salt and calcium in the cheese whey samples may have played key roles in enhancing treatment efficiency, but no conclusive evidence was traced to either. Future controlled studies could focus on the direct impact of salt and calcium addition on the treatment efficiency of PSW. Based on the salt concentrations and pH values in the CWW reactors, it appears that they were not limiting. The modified Gompertz model provided the best fit for the obtained methane production data where lag time decreased over cycles. The first order kinetic model indicated an increase in the hydrolysis rate in reactors containing CWW between the first and second cycles where a maximum was established. The 16S rRNA sequencing results indicated that the archaeal community was dominated by *Methanothrix* at 84.3–99.9%. *Petrimonas* genus attained the highest RA up to 38.9% in the first two cycles, and then it decreased to approximately 0% for all reactors. *Syntrophomonas* was highly abundant in PSW reactors reaching up to 36%. *Acinetobacter* was present mostly in CWW reactors with a RA reaching up to 56.5%. *Acinetobacter* appears to play a detrimental role in the treatment of CWW when actively present in feedstock, potentially through its ability to scavenge acetate at the start of the treatment process and thus hindering the syntrophic microbial development among the various anaerobic microbial communities.

## Supplementary Information

Supplementary Information.

## Data Availability

The datasets generated and/or analyzed during the current study are available in the National Center for Biotechnology Information (NCBI) repository: https://www.ncbi.nlm.nih.gov/Traces/study/?acc=PRJNA824252&o=acc_s%3Aa.
