# Supplementary material for: Investigating microbial dynamics and potential advantages of anaerobic co-digestion of cheese whey and poultry slaughterhouse wastewaters
Source: Sci Rep. 2022 Jun 22;12:10529. doi: 10.1038/s41598-022-14425-1 (PMC9217800; doi:10.1038/s41598-022-14425-1)
Supplement: Supplementary file 1 — Supplementary Information. [file 41598_2022_14425_MOESM1_ESM.docx]

**Supplementary Information**

**1. Theoretical Methane Potential**

The theoretical methane yield was determined in accordance with Buswell’s formula which accounts for lipid, protein and carbohydrate content of the organic matter expressed in Equation (S1) as mL-CH_4_/gVS_added_:

$\text{TMP}_{\text{Chem}\text{ical}\text{ Comp}\text{osition}}\text{= 415 × Carbohydrates }\left( \text{\%TS} \right)\text{+ 496 × Proteins }\left( \text{\%TS} \right)\text{+1014×lipids (\%TS)}$ (S1)

Eq. (S1) can be converted into added mass of COD based on the chemical composition of each substrate and the COD/VS ratio for these components ^1-3^.

**2. Mono- and Co-digestion Performance Criteria**

The biodegradability of the substrates, which compares the measured cumulative methane produced relative to the theoretical methane production in the different mixtures was assessed using Equation (S2):

$\text{Biodegradability }\left( \text{\%} \right)\text{=}\frac{\text{SMP}}{\text{TMP}}\text{×100}$ (S2)

Where SMP is the specific methane production (mL-CH_4_/g COD) and TMP is the theoretical methane production (mL CH_4_/g COD) of the utilized substrate mixture.

To assess the effects of co-digestion, a synergy factor is calculated based on the treatment efficiency. Synergy is defined as the ratio of the co-digestion efficiency of a mixed substrate over the corresponding weighted digestion efficiency of equivalent substrate using mono-digestion. The digestion efficiency is defined as the ratio of the measured methane produced over the methane potential based on the COD of the substrate ^4^. The synergy factors were determined according to Equation (S3):

$\text{Synergy factor =}\frac{\text{Co-digestion Efficiency}}{\text{Weighted Co-digestion Efficiency}}$ (S3)

Factor values greater than 1 indicate the presence of synergy while values less than 1 indicate reduced efficiency.

**3. Analytical Methods**

**3.1 Substrate properties**

TS, VS, TSS and VSS were measured according to APHA standard methods ^5^. The total and soluble COD, reactive phosphate, inorganic phosphate (meta-, poly-), total phosphate, ammonia, total organic carbon (TOC) and total nitrogen (TN), and TKN for soluble and total samples were all measured using HACH ‘s colorimetric techniques and HACH spectrophotometer (model DR3900) using the following methods in respective order: 8000, 8048, 8180, 8190, 10031, 10128, 10072 and 10242, respectively. Samples were centrifuged (Thermo Scientific Multifuge X1R) at 13000 rpm for 15 minutes and filtered using micro-filters (Kinesis KX Syringe Filters, Nylon 25mm, 0.22µm) to obtain the soluble content of samples. pH was measured daily for each reactor using a calibrated pH meter. Volatile fatty acid content (acetic, propionic and butyric acid) was measured using ion chromatography (Metrohm 882 Compact IC plus; using column 250/7.8 for measuring organic acids). Biogas produced from each reactor was measured on a daily basis using Tedlar gasbags of 10-L capacity with samples stored in 1-L Tedlar gas bags. CH_4_ and CO_2_ content was analyzed using gas chromatography (GC) (7890B Agilent Technologies, USA) with thermal conductivity detector (TCD). Sludge samples were extracted for microbial community analysis at the end of each experimental phase to monitor the variation in the community using 2-mL centrifuge tubes and stored in a freezer at -20°C. K^+^, Na^+^, Mg^2+^, Cl^-^, NO_2_^-^, NO_3_^-^, SO_4_^2-^ were quantified by ion chromatography using an 882 Compact IC Plus (Metrohm USA) equipped with a chemical suppressor module and conductivity detector. Cl^-^, NO_2_^-^, NO_3_^-^, SO_4_^2-^ ions were detected after separation using a Metrosep A supp 5 Column (250 x 4 mm) with an eluent consisting of 3.5 mmol. L^-1^ sodium carbonate and 1 mmol. L^-1^ sodium hydrogen carbonate, eluent flow rate of 0.7 mL.min-1, and suppressor regenerant of 0.1 mol. L^-1^ sulfuric acid. K^+^, Na^+^, Mg^2+^ ions were detected after separation using a Metrosep C 4 Column (150 x 4.0 mm) with an eluent consisting of 4mmol. L^-1^ nitric acid (69%) at a flow rate of 0.9 mL. min^-1^. Injection volumes of 20 µL were used for all measurements. Ca^2+^ was measured using a PerkinElmer PinnAAcle 900H atomic absorption spectrophotometer. The C/N ratios were obtained based on the total TOC and TKN values for the substrates ^6^.

The protein extraction was carried out using the ultrasonic-alkali method suggested by ^7^. Proteins’ content was determined by a bicinchoninic acid (BCA) protein assay (Pierce™ Kit, Thermo-Scientific). Carbohydrates extraction was achieved by a two-step digestion as described by ^8,9^. The total carbohydrates quantification was executed using a phenol- H_2_SO_4_ method (Total Carbohydrate Assay Kit, Sigma-Aldrich kit). Lipids extraction was performed according to the Bligh & Dyer process ^10^ with slight modifications. The lipids content was measured by weighing after solvent evaporation and drying until constant weight was obtained.

Table S1 - Agro-industrial wastewater characteristics.

| Parameter | Units | R1 | R2 | R3* | R4* | R5* | Inoculum |
| --- | --- | --- | --- | --- | --- | --- | --- |
| PSW Content | % | 0 | 100 | 25 | 75 | 50 |  |
| CWW Content | % | 100 | 0 | 75 | 25 | 50 |  |
| pH |  | 5.7 | 6.6 | 5.9 | 6.4 | 6.0 | 8.4 |
| TS | % | 9.8 | 7.9 | 9.3 | 8.3 | 8.8 | 1.3 |
| VS | % TS | 85.9 | 95.8 | 88.3 | 93.3 | 90.8 | 64.1 |
| Ash | % TS | 14.2 | 4.2 | 11.7 | 6.7 | 9.2 | 35.9 |
| Carbohydrates | % TS | 33.1 | 6.2 | 26.4 | 13.0 | 19.7 |  |
| Proteins | % TS | 52.4 | 25.9 | 45.8 | 32.5 | 39.1 |  |
| Lipids | % TS | 0.4 | 63.6 | 16.2 | 47.8 | 32.0 |  |
| COD/VS | g COD/g VS | 1.0 | 2.1 | 1.2 | 1.8 | 1.5 |  |
| tCOD | mg COD/L | 91300 | 86500 | 83200 | 86600 | 82700 | 29350 |
| sCOD | mg COD/L | 81500 | 16800 | 65800 | 27650 | 38550 | 433 |
| tTOC | mg C/L | 29900 | 15829 | 26382 | 19347 | 22864 |  |
| sTOC | mg C/L | 28800 | 4896 | 22824 | 10872 | 16848 |  |
| tTKN | mg TKN-N/L | 894 | 726 | 852 | 768 | 810 |  |
| sTKN | mg TKN-N/L | 826 | 328 | 702 | 453 | 577 |  |
| Total PO_4_^3-^ | mg PO_4_^3-^/L | 2880 | 127 | 2192 | 816 | 1504 |  |
| Organic Phosphate | mg PO_4_^3-^/L | 1245 | 22 | 939 | 328 | 634 |  |
| Inorganic Phosphate (meta-, poly-) | mg PO_4_^3-^/L | 425 | 0 | 319 | 106 | 213 |  |
| Reactive Phosphate | mg PO_4_^3-^/L | 1210 | 106 | 934 | 382 | 658 |  |
| Ammonia | mg NH_3_/L | 258 | 42 | 204 | 96 | 150 |  |
| Acetic Acid | mg/L | 417 | 376 | 444 | 381 | 405 |  |
| Propionic Acid | mg/L | 8 | 25 | 11 | 19 | 15 |  |
| Butyric Acid | mg/L | 0 | 0 | 0 | 0 | 0 |  |
| ^[[1]](#footnote-1)^C/N ratio |  | 33.4 | 21.8 | 30.54 | 24.72 | 27.63 |  |
| Ca^2+^ | mg/L | 556 | 2 | 417 | 140 | 279 |  |
| K^+^ | mg/L | 1482 | 151 | 1149 | 484 | 779 |  |
| Na^+^ | mg/L | 3011 | 409 | 2360 | 1059 | 1608 |  |
| Mg^2+^ | mg/L | 378 | 108 | 175 | 310 | 148 |  |
| Cl^-^ | mg/L | 5195 | 86 | 3918 | 1364 | 2619 |  |
| NO_2_^-^ | mg/L | 30 | 3 | 24 | 10 | 16 |  |
| NO_3_^-^ | mg/L | 2.4 | 0.4 | 1.9 | 0.9 | 1.3 |  |
| SO_4_^2-^ | mg/L | 149 | 32 | 120 | 61 | 82 |  |

*Characterization data were calculated from R1 and R2 except for pH.

Table S2 - Summary of kinetic modelling results (First order and modified Gompertz models) for all reactors during all cycles.

| Cycle | Parameter | Units | | | R1 | | R2 | R3 | R4 | R5 |
| --- | --- | --- | --- | --- | --- | --- | --- | --- | --- | --- |
| Acclimation | First Order Model |  | |  | |  | |  |  |  |
|  | Q_0_ | mL-CH_4_/g COD | | 234.139 | | 393.266 | | 276.503 | 367.700 | 335.781 |
|  | k | d^-1^ | | 0.251 | | 0.229 | | 0.146 | 0.258 | 0.141 |
|  | r^2^ | - | | 0.95 | | 0.95 | | 0.97 | 0.98 | 0.98 |
|  | Modified Gompertz Model |  | |  | |  | |  |  |  |
|  | Q_0_ | mL-CH_4_/gCOD | | 223.818 | | 362.536 | | 250.722 | 370.024 | 311.626 |
|  | P_m­_ | mL-CH_4_/gCOD/d | | 38.717 | | 60.446 | | 26.398 | 40.081 | 27.202 |
|  | λ | d | | 4.916 | | 12.912 | | 4.598 | 6.100 | 4.851 |
|  | r^2^ | - | | 0.98 | | 1.00 | | 0.99 | 0.99 | 0.99 |
| Cycle 1 | First Order Model |  | |  | |  | |  |  |  |
|  | Q_0_ | mL-CH_4_/gCOD | | 298.839 | | 380.000 | | 372.240 | 371.683 | 401.966 |
|  | k | d^-1^ | | 0.365 | | 0.217 | | 0.114 | 0.294 | 0.199 |
|  | r^2^ | - | | 0.99 | | 0.95 | | 0.97 | 0.98 | 0.98 |
|  | Modified Gompertz Model |  | |  | |  | |  |  |  |
|  | Q_0_ | mL-CH_4_/gCOD | | 329.350 | | 380.000 | | 406.626 | 350.125 | 532.797 |
|  | P_m­_ | mL-CH_4_/gCOD/d | | 39.282 | | 45.578 | | 28.949 | 60.330 | 36.062 |
|  | λ | d | | 1.095 | | 2.213 | | -0.140 | 1.326 | 1.183 |
|  | r^2^ | - | | 0.98 | | 0.99 | | 1.00 | 0.99 | 0.99 |
| Cycle 2 | First Order Model |  | |  | |  | |  |  |  |
|  | Q_0_ | mL-CH_4_/gCOD | | 294.798 | | 404.235 | | 300.456 | 314.369 | 307.706 |
|  | k | d^-1^ | | 0.404 | | 0.258 | | 0.409 | 0.638 | 0.393 |
|  | r^2^ | - | | 0.98 | | 0.95 | | 0.99 | 0.99 | 0.99 |
|  | Modified Gompertz Model |  | |  | |  | |  |  |  |
|  | Q_0_ | mL-CH_4_/gCOD | | 282.952 | | 373.958 | | 287.817 | 311.652 | 295.374 |
|  | P_m­_ | mL-CH_4_/gCOD/d | | 75.303 | | 96.207 | | 79.554 | 95.093 | 73.926 |
|  | λ | d | | -0.088 | | 0.711 | | -0.050 | 0.445 | -0.188 |
|  | r^2^ | - | | 1.00 | | 1.00 | | 1.00 | 1.00 | 1.00 |
| Cycle 3 | First Order Model |  |  | | |  | |  |  |  |
|  | Q_0_ | mL-CH_4_/gCOD | | 298.172 | | - | | 323.12 | 416.627 | 337.940 |
|  | k | d^-1^ | | 0.342 | | - | | 0.338 | 0.208 | 0.340 |
|  | r^2^ | - | | 0.98 | | - | | 1.00 | 0.94 | 1.00 |
|  | Modified Gompertz Model |  | |  | |  | |  |  |  |
|  | Q_0_ | mL-CH_4_/gCOD | | 285.537 | | - | | 307.561 | 345.843 | 327.832 |
|  | P_m­_ | mL-CH_4_/gCOD/d | | 77.403 | | - | | 64.594 | 93.925 | 59.431 |
|  | λ | d | | -0.365 | | - | | -0.35 | 0.791 | -0.736 |
|  | r^2^ | - | | 1.00 | | - | | 1.00 | 0.96 | 1.00 |
| Cycle 4 | First Order Model |  | |  | |  | |  |  |  |
|  | Q_0_ | mL-CH_4_/gCOD | | 349.091 | | - | | 337.088 | 427.622 | 329.860 |
|  | k | d^-1^ | | 0.291 | | - | | 0.333 | 0.249 | 0.340 |
|  | r^2^ | - | | 0.99 | | - | | 0.98 | 0.96 | 0.98 |
|  | Modified Gompertz Model |  | |  | |  | |  |  |  |
|  | Q_0_ | mL-CH_4_/gCOD | | 287.212 | | 380.000 | | 287.012 | 326.732 | 282.111 |
|  | P_m­_ | mL-CH_4_/gCOD/d | | 77.403 | | 55.502 | | 88.563 | 99.452 | 86.818 |
|  | λ | d | | 0.056 | | 1.636 | | 0.142 | 0.371 | 0.102 |
|  | r^2^ | - | | 1.00 | | 0.97 | | 1.00 | 1.00 | 1.00 |

Table S3 – Values for the kinetic parameters obtained from the first order model and modified Gompertz reported in the literature for similar feed composition

|  |  | Modified Gompertz Model | | | First Order Model | |
| --- | --- | --- | --- | --- | --- | --- |
| Literature | Substrate  Utilized | P_m_  (mL-CH_4_/gVS/d) | λ  (d) | r^2^ | k  (d^-1^) | r^2^ |
| Zahan et al. ^11^ | Yogurt Whey (2% VS) | 13.9 | 0.00 | 0.99 | 0.07 | 0.99 |
| Nielfa et al.^12^ | OFMSW^a^/biological sludge (91/9 %VS) | 35.1 | 0.31 | 0.99 | 0.23 | 0.99 |
| Neves et al.^13^ | Excess Lipid Content | - | - | - | 0.12 | - |
| Neves et al.^13^ | Excess Protein Content | - | - | - | 0.24 | - |
| Otero et al.^14^ | Slaughterhouse waste^b^ | 87.4^c^ | 1.81 | 0.99 | - | - |
| Bella and Rao ^15^ | 100%VS Cheese whey | 7.52 | 1.84 | - | - | - |
| Ware and Power ^16^ | Soft offal (SO) waste | 22.9 | 3.02 | 0.99 | - | - |

^a^ OFMSW: Organic fraction of municipal solid waste

^b^ Averaged for all waste streams excluding blood, sludge and manure

^c^ Reported on a per COD basis

R1

R2

R3

R4

R5

C0 (Acclimation)

C1

C4

C3

C2

Figure S1 - Daily methane production during the acclimation and 4 cycles for each of the reactors: R1 (100% CWW), R2 (100% PSW), R3 (75% CWW & 25% PSW), R4 (25% CWW & 75% PSW) and R5 (50% CWW & 50% PSW).

Figure S2 - pH variation during the acclimation and 4 cycles for each of the reactors: (a) R1 (100% CWW), (b) R2 (100% PSW), (c) R3 (75% CWW & 25% PSW), (d) R4 (25% CWW & 75% PSW) and (e) R5 (50% CWW & 50% PSW).

Figure S3- VFA profile distribution for acetic (●), propionic (●) and butyric (●) acid during the acclimation and 4 cycles for each of the reactors: (a) R1 (100% CWW), (b) R2 (100% PSW), (c) R3 (75% CWW & 25% PSW), (d) R4 (25% CWW & 75% PSW) and (e) R5 (50% CWW & 50% PSW).

Figure S4. Heat map representing the relative abundance of the of bacterial and archaeal communities in the wastewaters at the genus level and characterized by a relative activity of 1% or greater for Bacteria and 2% or greater for Archaea in at least one sample.

| **Bacterial and archaeal genera** | **Upper and lower left quadrants** | **Red and blue clusters** |
| --- | --- | --- |
| *Petrimonas* | 0.003 |  |
| *Proteiniphilum* | 0.04 |  |
| *Micropepsis* | 0.008 | 0.02 |
| *Nitrosospira* | 0.02 |  |
| *Cloacibacillus* | 0.001 |  |
| *Aminivibrio* | 0.001 | 0.006 |
| *Levilinea* | 0.006 |  |
| *Macellibacteroides* |  | 0.04 |
| *Acinetobacter* |  | 0.001 |
| *Soehngenia* |  | 0.02 |
| *Syntrophomonas* |  | 0.009 |
| *Methanomassiliicoccus* | 0.03 |  |
| *Methanospirillum* |  | 0.05 |

Table S4 - Clusters-based microbial comparison (P-values, unpaired t-test).

R5

R4

R3

R2

R1

C0 (Acclimation)

C1

C4

C3

C2

Figure S5 – Daily CO_2_ production during the acclimation and 4 cycles for each of the reactors: R1 (100% CWW), R2 (100% PSW), R3 (75% CWW & 25% PSW), R4 (25% CWW & 75% PSW) and R5 (50% CWW & 50% PSW).

**References**

1 Henze, M., van Loosdrecht, M. C., Ekama, G. A. & Brdjanovic, D. *Biological wastewater treatment*. (IWA publishing, 2008).

2 Mata-Alvarez, J., Macé, S. & Llabres, P. Anaerobic digestion of organic solid wastes. An overview of research achievements and perspectives. *Bioresource technology* **74**, 3-16 (2000).

3 Rittmann, B. E. & McCarty, P. L. *Environmental biotechnology: principles and applications*. (McGraw-Hill Education, 2001).

4 Ebner, J. H., Labatut, R. A., Lodge, J. S., Williamson, A. A. & Trabold, T. A. Anaerobic co-digestion of commercial food waste and dairy manure: Characterizing biochemical parameters and synergistic effects. *Waste management* **52**, 286-294 (2016).

5 APHA, A. WEF (2012) Standard methods for the examination of water and wastewater 22nd ed. *American Public Health Association, Washington*.

6 Haider, M. R., Yousaf, S., Malik, R. N. & Visvanathan, C. Effect of mixing ratio of food waste and rice husk co-digestion and substrate to inoculum ratio on biogas production. *Bioresource technology* **190**, 451-457 (2015).

7 Gao, J., Wang, Y., Yan, Y. & Li, Z. Ultrasonic-alkali method for synergistic breakdown of excess sludge for protein extraction. *Journal of Cleaner Production* **295**, 126288 (2021).

8 Martens, D. A. & Loeffelmann, K. L. Improved accounting of carbohydrate carbon from plants and soils. *Soil Biology and Biochemistry* **34**, 1393-1399 (2002).

9 Pleissner, D., Lam, W. C., Sun, Z. & Lin, C. S. K. Food waste as nutrient source in heterotrophic microalgae cultivation. *Bioresource technology* **137**, 139-146 (2013).

10 Bligh, E. G. & Dyer, W. J. A rapid method of total lipid extraction and purification. *Canadian journal of biochemistry and physiology* **37**, 911-917 (1959).

11 Zahan, Z., Othman, M. Z. & Muster, T. H. Anaerobic digestion/co-digestion kinetic potentials of different agro-industrial wastes: A comparative batch study for C/N optimisation. *Waste Management* **71**, 663-674 (2018).

12 Nielfa, A., Cano, R. & Fdz-Polanco, M. Theoretical methane production generated by the co-digestion of organic fraction municipal solid waste and biological sludge. *Biotechnology Reports* **5**, 14-21 (2015).

13 Neves, L., Goncalo, E., Oliveira, R. & Alves, M. Influence of composition on the biomethanation potential of restaurant waste at mesophilic temperatures. *Waste management* **28**, 965-972 (2008).

14 Otero, A., Mendoza, M., Carreras, R. & Fernandez, B. Biogas production from slaughterhouse waste: Effect of blood content and fat saponification. *Waste Management* **133**, 119-126 (2021).

15 Bella, K. & Rao, P. V. Anaerobic co-digestion of cheese whey and septage: Effect of substrate and inoculum on biogas production. *Journal of Environmental Management* **308**, 114581 (2022).

16 Ware, A. & Power, N. Modelling methane production kinetics of complex poultry slaughterhouse wastes using sigmoidal growth functions. *Renewable Energy* **104**, 50-59 (2017).

1. [↑](#footnote-ref-1)
